# Introduction of a Brain MRI Scoring System with Clinical Relevance for Sturge-Weber Syndrome

**DOI:** 10.1016/j.acra.2026.02.021

**Published:** 2026-03-03

**Authors:** Csaba Juhász, Aimee F. Luat, Michael E. Behen, Ajay Kumar

**Affiliations:** Department of Pediatrics, Wayne State University School of Medicine, 400 Mack Ave., Detroit, Michigan 48201 (C.J., A.F.L., M.E.B., A.K.); Department of Neurology, Wayne State University School of Medicine, Detroit, Michigan (C.J., M.E.B.); Translational Imaging Laboratory, Wayne State University School of Medicine, University Health Center, Detroit, Michigan (C.J., M.E.B.); Children’s Hospital of Michigan, Detroit, Michigan (A.F.L.); Department of Pediatrics, Central Michigan University, Detroit, Michigan (A.F.L.); Division of Neuroradiology, Perelman School of Medicine, University of Pennsylvania, 3400 Spruce St., Philadelphia, Pennsylvania 19104 (A.K.).

**Keywords:** Sturge-Weber syndrome, Magnetic Resonance Imaging, Calcification, Atrophy, Cognitive Functions

## Abstract

**Rationale and Objectives:**

Sturge-Weber syndrome (SWS) is a sporadic neurocutaneous disorder marked by cerebral venous abnormalities, progressive parenchymal damage, and early-onset neuro-cognitive complications. Existing imaging assessments lack standardized, quantitative approaches to capture the full disease burden. Here we tested an magnetic resonance imaging (MRI)-based scoring system that comprehensively captures both vascular and parenchymal brain abnormalities in SWS.

**Materials and Methods:**

Twenty-five young patients (mean age, 9.5 years; range, 1–24 years) with unilateral SWS brain involvement underwent 3 T MRI using a standardized protocol (with pre- and post-contrast sequences) and formal neuro-cognitive evaluation. Six imaging features, four vascular and two parenchymal, were scored by two investigators across lobes using a 3-point scale. Interrater reliability was assessed using intra-class correlation coefficients (ICC), and associations with neuro-cognitive variables were tested using Spearman’s rank correlations.

**Results:**

Both the total MRI score and each MRI subscore demonstrated excellent interrater reliability (ICC range: 0.91–0.99). Motor functions showed strong inverse correlations with the total MRI scores (ρ = −0.82, *p* < 0.0001). Low verbal IQ correlated with extensive calcifications (ρ = −0.55, *p* < 0.01). High seizure frequency correlated with greater pial enhancement (*p* < 0.05) and choroid plexus scores (*p* < 0.01). The new multiparametric score outperformed a previously established asymmetry-based MRI score in its associations with cognitive outcomes and seizure frequency.

**Conclusion:**

This reliable and user-friendly MRI scoring system, that integrates multiple vascular and parenchymal features relevant to SWS pathophysiology, can be highly suitable for longitudinal monitoring, prognostication, and standardized outcome assessment in multicenter research and therapeutic trials.

## INTRODUCTION

Sturge–Weber syndrome (SWS) is a rare, sporadic neurocutaneous disorder caused by a somatic activating mutation in the GNAQ gene during embryonic development, although some phenotypes have also been associated with GNA11 gene variants (1,2). SWS is characterized by a triad of features: facial capillary malformations (port-wine birthmark), leptomeningeal venous malformation (affecting one hemisphere in the vast majority of cases), and ocular involvement—most commonly vascular glaucoma (present in about 50% of SWS patients). Neurological manifestations are often the most disabling and include seizures, hemiparesis, visual field deficits, and variable cognitive impairment. These symptoms typically begin in early childhood and progress variably among affected individuals (3–7).

Magnetic resonance imaging (MRI) plays a crucial role in diagnosing and monitoring brain involvement longitudinally in SWS. Classic MRI findings include leptomeningeal (pial) enhancement overlying the affected hemisphere, reflecting abnormal leptomeningeal venous vasculature. Additional findings—such as cortical atrophy, parenchymal calcifications, gray- and white-matter volume loss, prominent deep medullary veins, choroid plexus enlargement, and absent deep cerebral or basal veins—likely reflect chronic venous insufficiency, ischemic injury, and compensatory vascular remodeling (8–12). These abnormalities are clinically meaningful and can guide both surgical planning and prognostic assessment (13).

Despite their diagnostic importance, structured and integrative imaging approaches that quantify overall disease burden in SWS remain limited (14–17). Imaging assessments often rely primarily on qualitative or binary descriptions and tend to focus on isolated features, most often pial enhancement or cortical atrophy, without accounting for the broader and complex interplay of vascular and parenchymal abnormalities (9,11,18,19). This limits both cross-patient comparisons and the ability to objectively track disease progression or treatment response over time.

Recent collaborative efforts have highlighted the need for standardized, quantitative MRI-based phenotyping and the development of imaging biomarkers to enhance prognostication and facilitate therapeutic trials in SWS (17,20). A reproducible, multiparametric scoring system that integrates various vascular and parenchymal abnormality features into a unified framework could address this unmet need.

To overcome the above limitations, in this study we introduce a novel MRI scoring system designed to comprehensively capture structural brain abnormalities in SWS, incorporating six imaging features—four vascular (pial enhancement, enlarged medullary veins, choroid plexus enlargement, and absence of deep/basal cerebral veins) and two parenchymal (cortical atrophy and parenchymal calcification)—scored across cerebral lobes.

The objectives of this study were twofold: (i) to assess the interrater reliability of this MRI scoring system across experienced raters; and (ii) to evaluate correlations between MRI-derived scores and key clinical outcomes—motor function, cognitive performance, and seizure severity—i.e., indicators of disease burden in patients with SWS.

## MATERIALS AND METHODS

### Study Population

Participants were selected from a prospective study conducted between 2010 and 2024. Inclusion criteria were: (i) age 1–24 years; (ii) previous diagnosis of unilateral SWS brain involvement confirmed by MRI; (iii) availability of high-quality precontrast and postcontrast 3 T MRI from the same scanner; and (iv) no prior brain surgery. Patients older than 30 months underwent formal neuropsychologic testing. The study was approved by the Human Investigation Committee (#123500MP4F and #IRB-20–01–1765). Written informed consent was obtained from parents or participants, and written assent from children aged 13–18 years.

### Clinical Assessment and Cognitive Evaluation

SWS diagnosis was established clinically and radiologically by neurologists (A.F.L. and C.J.). Seizure history and variables (age at onset, duration, frequency) was extracted from medical records and parent interviews. Epilepsy severity was graded using a previously reported seizure frequency scale (11): 0 = none in past year; 1 = 1–11/year; 2 = 1–4/month; 3 = ≥4/month. Age-appropriate neuropsychologic testing (Wechsler scales) provided verbal, nonverbal intelligence quotient (VIQ, NVIQ, respectively) as well as full-scale IQ, and fine-motor function was assessed via the Purdue Pegboard (age < 5 years) or the Grooved Pegboard Test (age 5 years and above), all performed within 24 h of the prospective MRI.

### MRI Acquisition and Processing

A Siemens MAGNETOM Verio 3 T scanner (Siemens Healthineers, Erlangen, Germany) was used to scan all participants. The MRI acquisition protocol included: (i) an axial T2-weighted turbo spin echo image (4 mm slice thickness, echo time [TE]/repetition time [TR]: 93/6000 ms; acquisition time: 1 min 14 s); (ii) an axial fluid attenuated inversion recovery (2 mm slice thickness, TE/TR: 128/9000 ms; 3 min 56 s); (iii) susceptibility-weighted imaging (SWI) (voxel size 0.5 × 0.5 × 2.0 mm^3^; field of view 224 × 168 × 128 mm; base resolution 448; TE 5.1ms/18ms, TR 30ms/30 ms; bandwidth 160~410 Hz/pixel, 2 × accelerated parallel imaging with 24 reference lines, and 6/8 partial Fourier along phase encoding; 5 min); and (iv) a volumetric axial T1-weighted 3-dimensional magnetization prepared rapid gradient echo (voxel size: 0.9 × 0.9 × 0.9 mm^3^, TE/TR: 3/1700 ms; 4 min 35 s). This latter sequence was repeated after the administration of gadobutrol (0.1 mL/kg) as a contrast agent. Young patients underwent moderate sedation during MRI scanning in order to minimize motion artifacts.

### MRI Scoring System Design

**Figure 1.**
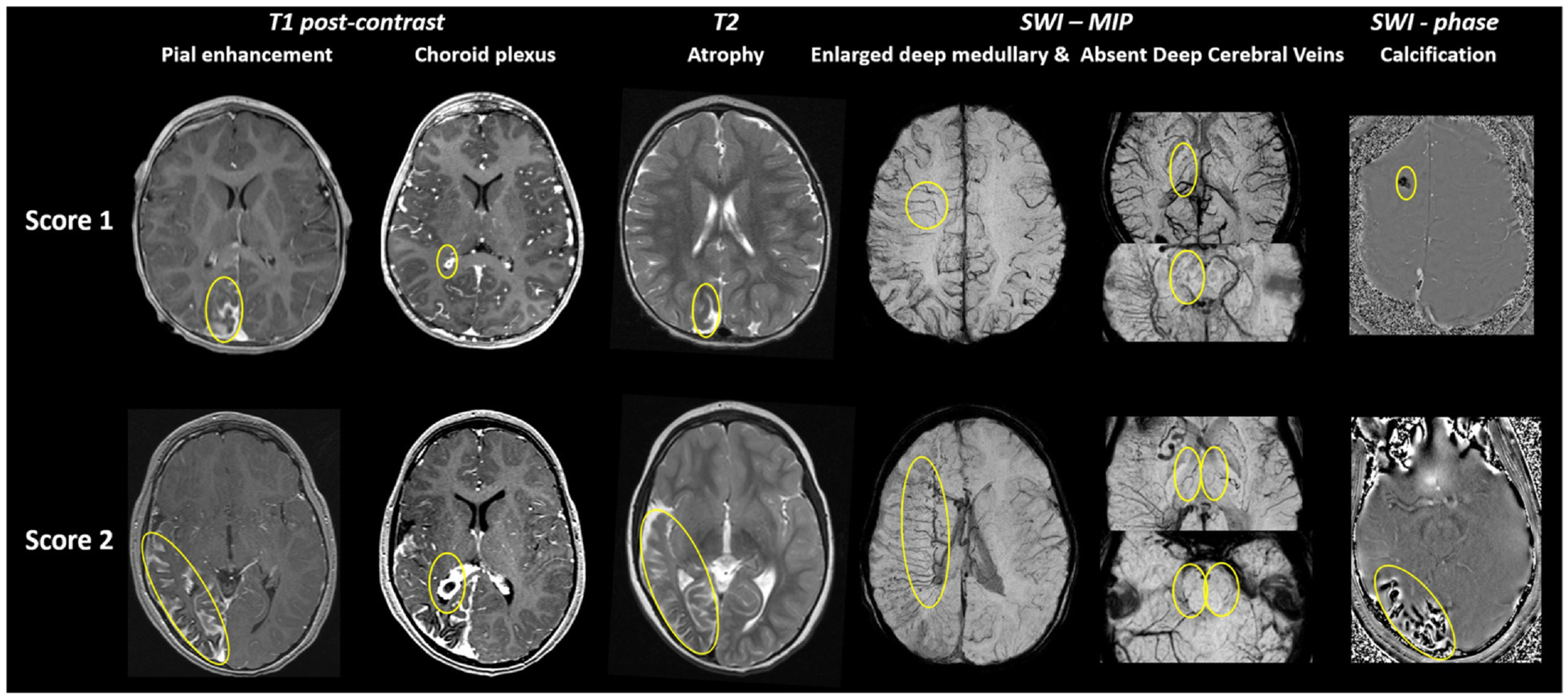
Relevant MRI sequences used for MRI scoring, showing venous vascular and parenchymal abnormalities, with examples of scores 1 and 2 for each feature (abnormalities are circled). Pial enhancement: sublobar (score 1), extensive lobar (score 2); choroid plexus: mild asymmetry (score 1) vs. robust asymmetry (score 2); atrophy: sublobar (score 1) vs. extensive lobar (score 2); enlarged deep medullary veins: sublobar (score 1) vs. extensive lobar (score 2); absent deep cerebral veins: unilateral absence of the internal cerebral vein (upper panel) and the basal vein of Rosenthal (lower panel) vs. bilateral absence of the same veins (score 2); calcification: small sublobar (score 1) vs. extensive lobar (score 2).

Each MRI was scored across six imaging features known to be relevant in SWS pathology, using MRI sequences listed in Table 1. The subscores included four intracranial vascular abnormalities: (1) pial enhancement, (2) enlarged medullary veins, (3) choroid plexus enlargement, and (4) absence of deep cerebral veins (internal cerebral vein and basal vein of Rosenthal), as well as two brain parenchymal abnormalities: (5) cortical atrophy, and (6) parenchymal calcification (Figure 1). Features 1,2,5 and 6 were scored in each cerebral lobe (frontal, parietal, temporal, occipital) using a 3-point ordinal scale: 0 = none / normal, 1 = mild / focal, and 2 = extensive / severe (Table 1). Choroid plexus enlargement in the posterior horn of the ventricle was scored hemispherically only. Absence of the internal cerebral vein and basal vein can be both unilateral and bilateral (even in otherwise unilateral SWS) (10), and therefore, these were scored accordingly (Table 1). Total MRI scores were calculated by summing all feature subscores, yielding a maximum potential score of 38.

MRI features were also scored using previously reported SWS MRI scoring criteria (21), modified from an original system (22) and based upon T1 pre- and postcontrast MRI images, as follows: [1] no asymmetry, [2] mild asymmetry (atrophy or angiomatosis only), [3] moderate asymmetry (angiomatosis and mild atrophy), and [4] severe asymmetry (angiomatosis and severe atrophy). Scores were assigned in four brain regions (frontal, temporal, parietal, occipital lobes) and summed up to give the MRI asymmetry score (with possible scores ranging from 4 to 16).

A board-certified neuroradiologist (A.K.) and a neurologist with extensive SWS imaging experience (C.J.) independently scored all scans. After initial scoring, consensus meetings resolved any discrepancies to create the final consensus scores, which were used for clinical correlations.

### Statistical Analysis

Normality was assessed by the Shapiro-Wilk test. Since several variables did not show normal distribution, non-parametric tests were used: Spearman’s ρ for correlations and the Mann–Whitney U test for group differences. Interrater reliability was evaluated using intraclass correlation coefficients (ICCs). To control inflated alpha in the testing of multiple correlations, a false discovery rate (FDR) procedure was employed (23). Both unadjusted and FDR-adjusted p-values are reported. To evaluate whether our available sample size provided sufficient power to detect intraclass correlations that can be considered at least good (i.e., ICC≥0.75 (24)), we have performed a post hoc power analysis, with the available sample size, ICC of 0.75, and a two-tailed α of 0.00625 (i.e., 0.05 Bonferroni-corrected due to eight different MRI scores assessed). Analyses used SPSS 30.0 (IBM, Armonk, NY), and the significance was set at p < 0.05.

## RESULTS

### Clinical and MRI variables

Twenty-five young patients (17 females; mean age 9.5 years (range: 1–24), 14 with right and 11 with left hemispheric brain involvement were included in the study. Clinical variables are summarized in Table 2. Twenty-four patients had a history of epilepsy. Motor T-scores were available for 21 patients, and Wechsler-based IQ scores were available for 20 patients.

### MRI Scores and Their Interrater Reliability

The mean, median and minimum/maximum values of the consensus MRI scores are listed in Table 3. Interrater reliability was excellent for both the MRI subscores and the total MRI score, with an ICC ranging from 0.913–0.995 (all p < 0.001; Supplementary table). The post-hoc power analysis indicated a power of 0.97, confirming to have excellent power to detect an ICC of 0.75 or above.

### Clinical Correlations of the MRI Scores

**Figure 2.**
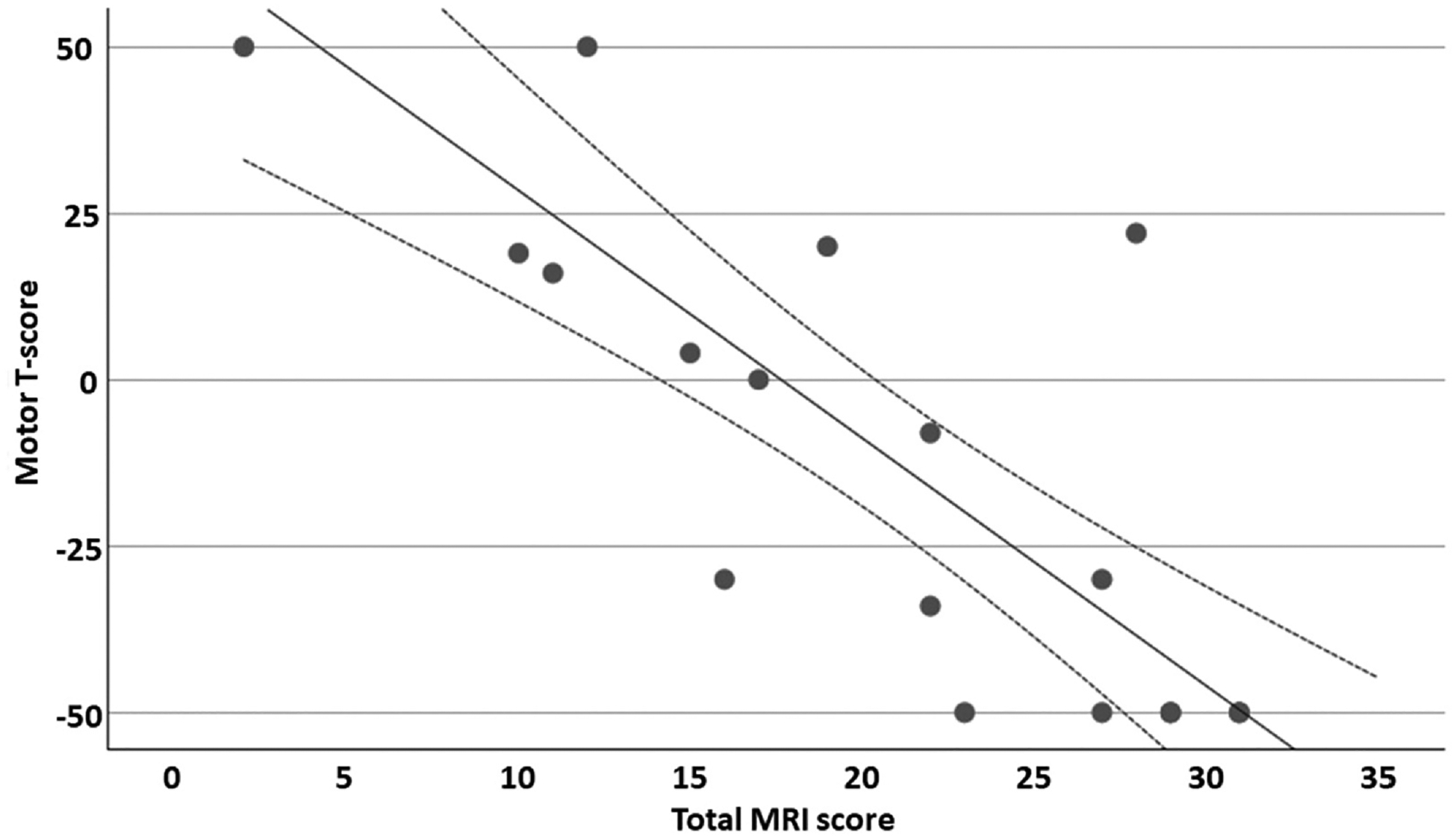
Strong inverse correlation between total MRI scores and motor T-scores. The graph includes a linear regression line with 95% confidence intervals (dotted lines) created with 21 data points (with 4 overlapping value pairs). Spearman’s rho was −0.82 (p=0.000006).

None of the clinical or MRI variables showed significant differences between left and right-sided SWS patients (p > 0.1 in all comparisons); therefore, all correlations included the whole group. The motor T-scores were closely correlated with both the total MRI scores and several subscores (including extent of pial enhancement, atrophy, and calcification; see Table 4), with the total scores showing the highest correlation coefficient (Spearman’s rho: 0.82, p=0.000006 without FDR correction) (Figure 2).

Among the clinical seizure variables, the seizure frequency scores showed correlations with both the pial enhancement and choroid plexus scores (p=0.013 and p=0.004, respectively), with only the latter surviving the FDR correction. VIQ scores showed the closest correlation with calcification scores and slightly weaker correlations with the atrophy and total scores (Table 4); full-scale IQ showed similar correlations, while NVIQ was not predicted significantly by any MRI variable, although both the total score and atrophy score showed a strong trend (p=0.06 for both, without FDR correction).

The previously reported MRI score, which relies on the combination of pial enhancement and atrophy (21), performed worse than the current total MRI score in terms of correlation with the motor score. Also, it showed no significant correlations with IQ scores or seizure variables in this cohort. No significant associations were found between any MRI score and age, age at onset, or duration of epilepsy.

## DISCUSSION

This study presents a novel, reliable, and practical MRI-based scoring system that comprehensively integrates multiple vascular and parenchymal imaging features of SWS. The system demonstrated outstanding reproducibility and also robust associations with motor and cognitive functions, making it well-suited for both clinical implementation and research applications. Pending further validation, adapting this MRI scoring system in standardized reporting templates could facilitate a more uniform assessment of the type, extent, and severity of vascular and parenchymal abnormalities in patients with SWS.

Prior MRI scoring schemes in SWS have been scarce and focused on leptomeningeal enhancement and the presence/extent of atrophy (18,21,22,25). One such hemispheric MRI asymmetry score (18) showed a good correlation with hemiparesis but only a weak association with cognitive outcomes, which were clinically assessed without formal testing. Other studies have reported associations between MRI scores and electroencephalography asymmetries (21) or composite neurological scores (25), but their limited feature sets and lack of utilization of SWI hindered their broader applicability. A previous study, comparing the utility of post-contrast T1 images and SWI employed a hemispheric scoring system (8). While the study showed the complementary nature of these MRI sequences, it did not assess the lobar extent of these abnormalities and did not establish clinical correlates.

In a recent study (19), a more comprehensive scoring system for multiple MRI features was employed in noncontrast MRI of SWS children; however, a binary scoring system was used in a small cohort (n = 10), which precluded meaningful MRI/clinical correlations. Another recent study (10) demonstrated that the absence of deep veins is common in SWS. Therefore, the present scoring system expanded this framework by incorporating additional, clinically relevant features, such as venous remodeling and calcification, thereby maximizing the diagnostic value of standard MRI sequences.

The integration of these features yielded a total MRI score that strongly correlated with motor functions, moderately outperforming previous scoring methods. The strong correlation between the MRI scores and motor deficits highlights the score’s ability to reflect cumulative disease burden. Parenchymal brain damage, including atrophy and calcification also showed strong associations with motor impairment. These abnormalities have been recognized as imaging markers of chronic parenchymal damage in SWS, typically reflecting progressive and irreversible changes. These features can be easily identified using standard MRI protocols that include SWI, enhancing the score’s clinical practicality.

Cognitive outcomes also showed correlations with the extent of parenchymal damage, although these correlations were less robust than those for motor function. This may reflect the multifactorial underpinnings of cognitive function in SWS, including the effects of seizure burden, age at onset, drug effects, and the capacity of early functional reorganization, particularly for speech (26,27).

Interestingly, only some vascular, not parenchymal, subscores showed significant positive correlations with seizure frequency scores. Most patients in this study had only mild/moderate epilepsy severity, with only one patient having at least weekly seizures (score 3). Future studies could determine if this finding is reproducible in cohorts that include more subjects with frequent seizures. Age and duration of epilepsy showed no associations with MRI variables, likely reflecting the fact that, while some abnormalities progress over time, the most robust progression of brain damage may occur during the first 3 years of life (28), while the vast majority (20/25) of the patients were above 3 years of age.

From a translational perspective, our scoring system offers several advantages. It is straightforward to implement, requires no advanced post-processing, and relies solely on routine MRI sequences. The use of a structured ordinal scale facilitates consistent scoring across institutions and readers, while the regional lobe-based approach enables detailed spatial mapping of disease burden and nuanced tracking of disease topography. These features make the tool highly adaptable for diverse clinical and research settings.

Looking ahead, the role of imaging biomarkers in SWS is poised to expand, particularly as novel therapies, including disease-modifying approaches, are expected to enter clinical trials. While operator-independent image-analytic approaches incorporating machine learning approaches are also being tested in SWS imaging (20), semi-quantitative tools like this scoring system will remain instrumental in the near future for stratifying patients, monitoring treatment effects, and evaluating disease progression in a standardized manner.

### Study limitations

Our study has some limitations, including a small sample size and a wide age range. SWS is a relatively rare condition, and large sample sizes would require very long data acquisition or a multicenter design. On the other hand, a single-center study with standardized imaging and neuro-psychology evaluations makes the data more uniform. While this study provided preliminary evidence for the clinical relevance of the MRI scores, a larger sample size, allowing multivariate statistics will be required to fully validate the clinical correlates of this scoring system. The interrater reliability will need to be also reassessed in multicenter data sets involving more heterogeneous MRI data. It should also be noted that the applied MRI scoring criteria may not be as effective in young infants, especially those under 1 year of age. Very young SWS patients may show additional or different imaging abnormalities from older patients, including accelerated myelination (29), lack of pial enhancement (12,30), or early diffusion abnormalities potentially linked to epileptogenesis (31), features that are less typical or less explored in later disease stages. Cortical malformations have also been reported in SWS, however, they are often difficult to distinguish from other SWS parenchymal abnormalities and, therefore, cannot be readily detected by MRI (32). Finally, this study employed a cross-sectional design, with MRI and clinical variables collected simultaneously. Longitudinal studies will need to evaluate the ability of these scores to predict future neuro-cognitive outcomes.

## CONCLUSIONS

This study introduces a simple, reproducible, and clinically relevant MRI scoring system for quantifying brain involvement in Sturge–Weber syndrome. By integrating vascular and parenchymal abnormalities into a unified framework, the system bridges the gap between structural imaging and functional outcomes. Its high reliability, ease of implementation, and adaptability position it as a valuable tool for patient monitoring, prognostication, and multicenter clinical research; however, further validation in multi-institutional datasets is warranted.

## Supplementary Material

1

## Figures and Tables

**TABLE 1.** Detailed MRI Scoring System for Vascular and Parenchymal Abnormalities (Scores of 0, 1, 2 for Each Imaging Characteristic, with a Possible Total Score of 0–38)

	Preferred Sequence (s)	Score 0	Score 1	Score 2	Score Range	No. of Sites	Full Score Range
*Intracranial vascular abnormalities*							
Pial enhancement (venous malformation)	Post-GAD T1 (or FLAIR)	No abnormal pial enhancement	Limited extent of abnormal pial enhancement *(affecting* < *50% of the lobe)*	Extensive abnormal pial enhancement *(affecting* ≥*50% of the lobe)*	0–2	4 lobes	0–8
Enlarged deep medullary veins	SWI MIP	Nonvisible or symmetric small med. veins	Limited extent of enlarged/asymmetric medullary veins *(affecting* < *50% of the lobe)*	Extensive enlarged/asymmetric medullary veins *(affecting* ≥*50% of the lobe)*	0–2	4 lobes	0–8
Enlarged choroid plexus (in posterior horn of the lateral ventricle)	Post-GAD T1 (or FLAIR, native or GAD)	Symmetric < *10% max diameter difference*	Mild asymmetry *10%*- < 50% *of max. diameter difference*	Robust asymmetry > *50% of max. diameter difference*	0–2	1	0–2
Absent internal cerebral vein (ICV)	SWI MIP	ICV present	ICV absent on 1 side	ICV is absent on both sides	0–2	1	0–2
Absent basal vein of Rosenthal (BVR)	SWI MIP	BVR present	BVR is absent on 1 side	BVR is absent on both sides	0–2	1	0–2
*Brain parenchymal abnormalities*							
Atrophy	T1 & T2	Symmetric gyri, sulci	Obvious atrophy with enlarged sulci, limited extent *(affecting* < *50% of the lobe)*	Obvious atrophy with enlarged sulci, extensive *(affecting* ≥*50% of the lobe)*	0–2	4 lobes	0–8
Calcification	SWI phase (dark or hypointense)	No signs of calcification	Limited extent of calcification *(affecting* <*50% of the lobe)*	Extensive calcification *(affecting* ≥*50% of the lobe)*	0–2	4 lobes	0–8

**TABLE 2.** Clinical Data of the Patients

	N	Minimum	Maximum	Mean	Median
Age	25	1.0	24.0	9.5	9.3
Epilepsy duration (y)	24	0.7	23.4	8.6	6.7
Age at epilepsy onset (y)	24	0.1	7.0	1.0	0.6
Seizure frequency score	25	0	3	1.2	1.0
Motor T-score	21	−50	60	−12	−30
VIQ	20	60	118	91	89
NVIQ	20	60	102	82	82
FSIQ	20	60	107	86	91

VIQ, verbal intelligence quotient; NVIQ, nonverbal intelligence quotient; FSIQ, full-scale intelligence quotient.

Motor T-scores were not available for four subjects, and IQ values were not available for five subjects due to low age or lack of cooperation. One patient had no history of seizures.

**TABLE 3.** Consensus MRI Scores in the 25 Patients

	Minimum	Maximum	Mean	Median
Pial enhancement	0	8	4.8	6
EDMV	0	8	4.5	4
Choroid plexus	0	2	1.6	2
ICV absence	0	2	1.3	1
BVR absence	0	1	0.6	1
Atrophy	0	8	3.9	4
Calcification	0	8	3.2	3
*Total MRI score*	2	31	19.8	22

EDMV, enlarged deep medullary veins; ICV, internal cerebral vein; BVR, basal vein of Rosenthal.

**TABLE 4.** Spearman’s Rank Correlations Between MRI Scores and Clinical Variables

	Age	Seizure onset age	Epilepsy duration	Seizure frequency	Motor score	VIQ	NVIQ	FSIQ
MRI scores:	Spearman’s rho values (p values)							
*Pial enhancement*	−0.16	−0.28	−0.19	0.49 (p=0.013)	−**0.78 (p < 0.001)**	−0.36	−0.40	−0.39
*EDMV*	0.04	0.21	0.02	0.21	−0.33	0.03	−0.17	−0.05
*Choroid plexus*	0.06	−0.12	0.06	**0.55 (p=0.004)**	−0.47 (p=0.03)	−0.36	−0.41	−0.44
*ICV absent*	0.04	0.09	0.05	0.40 (p=0.049)	−0.32	−0.15	−0.37	−0.20
*BVR absent*	0.14	0.27	0.1	0.10	−0.35	−0.22	0.01	0.10
*Atrophy*	0.11	−0.38	0.10	0.32	−**0.76 (p < 0.001)**	−0.5 (p=0.023)	−0.42	−0.54 (p=0.014)
*Calcification*	0.16	−0.23	0.15	0.28	−**0.81 (p < 0.001)**	−**0.55 (p=0.010)**	−0.36	−0.53 (p=0.015)
*Total score*	0.08	−0.07	0.14	0.36	−**0.82 (p < 0.001)**	−0.45 (p=0.044)	−0.42	−0.48 (p=0.033)
Hatfield score	0.03	−0.33	0.02	0.38	−**0.78 (p < 0.001)**	−0.40	−0.37	−0.42

Significant p values (< 0.05) are indicated in parentheses. *P*≤*0.01 remained significant after false discovery rate correction, and these values are in bold font*.

## Take-home message

This study introduces a brain MRI scoring system integrating vascular and parenchymal features of Sturge-Weber syndrome. The scores have excellent interrater reliability, are associated with motor and cognitive abnormalities, and may be a valuable tool for monitoring disease progression and utilization in future clinical trials in patients with Sturge-Weber syndrome.

## APPENDIX A. SUPPORTING INFORMATION

Supplementary data associated with this article can be found in the online version at doi:10.1016/j.acra.2026.02.021.

## Abbreviations:

BVR: basal vein of Rosenthal
CI: confidence interval
EDMV: enlarged deep medullary veins
FDR: false discovery rate
FSIQ: full scale intelligence quotient
ICC: intraclass correlation coefficient
ICV: internal cerebral vein
MRI: magnetic resonance imaging
NVIQ: nonverbal intelligence quotient
SWI: susceptibility-weighted imaging
SWS: Sturge-Weber syndrome
TE: echo time
TR: repetition time
VIQ: verbal intelligence quotient
