# Supplementary material for: Introduction of a Brain MRI Scoring System with Clinical Relevance for Sturge-Weber Syndrome
Source: Acad Radiol. Author manuscript; Available in PMC 2026 Jun 12. (PMC13261762; doi:10.1016/j.acra.2026.02.021)
Supplement: 1 [file NIHMS2183848-supplement-1.docx]

**Supplementary table**. Intraclass correlation coefficients (ICC), with 95% Confidence Intervals (95%CI), of the MRI subscores and the total MRI score, as assessed by two investigators.

|  | **ICC** | **95%CI** | **p value** |
| --- | --- | --- | --- |
| **Pial enhancement** | 0.975 | 0.938-0.990 | <0.001 |
| **EDMV** | 0.975 | 0.939-0.990 | <0.001 |
| **Choroid plexus** | 0.957 | 0.894-0.983 | <0.001 |
| **Absent ICV** | 0.927 | 0.841-0.967 | <0.001 |
| **Absent BVR** | 0.913 | 0.809-0.961 | <0.001 |
| **Atrophy** | 0.968 | 0.920-0.987 | <0.001 |
| **Calcification** | 0.995 | 0.987-0.998 | <0.001 |
| ***Total score*** | 0.992 | 0.982-0.997 | <0.001 |

EDMV: Enlarged deep medullary veins; ICV: Internal cerebral vein; BVR: Basal vein of Rosenthal
